# Cellular signalling protrusions enable dynamic distant contacts in spinal cord neurogenesis

**DOI:** 10.1242/bio.061765

**Published:** 2025-01-21

**Authors:** Joshua Hawley, Robert Lea, Veronica Biga, Nancy Papalopulu, Cerys Manning

**Affiliations:** Faculty of Biology Medicine and Health, The University of Manchester, Manchester M13 9PT, UK

**Keywords:** Protrusions, HES5, Lateral inhibition, Spatial pattern, Notch, Spinal cord

## Abstract

In the developing mouse ventral spinal cord, HES5, a transcription factor downstream of Notch signalling, is expressed as evenly spaced clusters of high HES5-expressing neural progenitor cells along the dorsoventral axis. While Notch signalling requires direct membrane contact for its activation, we have previously shown mathematically that contact needs to extend beyond neighbouring cells for the HES5 pattern to emerge. However, the presence of cellular structures that could enable such long-distance signalling was unclear. Here, we report that cellular protrusions are present all along the apicobasal axis of individual neural progenitor cells. Through live imaging, we show that these protrusions dynamically extend and retract reaching lengths of up to ∼20 µm, enough to extend membrane contact beyond adjacent cells. The Notch ligand DLL1 was found to colocalise with protrusions, further supporting the idea that Notch signalling can be transduced at a distance. The effect of protrusions on the HES5 pattern was tested by reducing the density of protrusions using the CDC42 inhibitor ML141, leading to a tendency to decrease the distance between high HES5 cell clusters. However, this tendency was not significant and leaves an open question about their role in the fine-grained organisation of neurogenesis.

## Teaser

**Summary:** This study reveals dynamic cellular protrusions in mouse spinal cord neural progenitors that extend Notch signalling beyond adjacent cells, possibly contributing to periodic HES5 expression patterns during neurogenesis.

## INTRODUCTION

The central nervous system's (CNS) function depends upon spatial patterning to generate the correct cell types in the right place during embryogenesis. Previous studies have shown that morphogen gradients and transcription factor (TF) networks underlie spatial patterning by mediating the subdivision of the embryonic spinal cord in distinct but broad progenitor subdomains (Briscoe et al., 2000; Cohen et al., 2013; Ericson et al., 1995). Such subdomains generate distinct neuronal subtypes along the dorsoventral axis, for example, motor neurons ventrally and various types of interneurons more dorsally (Sagner and Briscoe, 2019).

Using a live protein reporter for the Notch target TF HES5, we have previously shown that a finer spatiotemporal organisation exists within the ventral interneuron neural progenitor domain. We previously reported that the oscillatory expression of HES5 over time is synchronised in microclusters of 3-7 cells in total. In the dorsoventral direction, microclusters span 2-3 cells in width, and these alternate between high and low clusters to form a spatially periodic pattern of expression. Furthermore, the pattern is highly dynamic and HES5 microclusters switch between high and low expression on the timescale of 7 h (Biga et al., 2021). Based on experimentation and modelling, we hypothesised that this fine-grained spatiotemporal pattern controls the rate and spacing of neurogenesis and that Notch-mediated self-organisation is sufficient to generate it (Biga et al., 2021; Hawley et al., 2022).

**Fig. 1.**
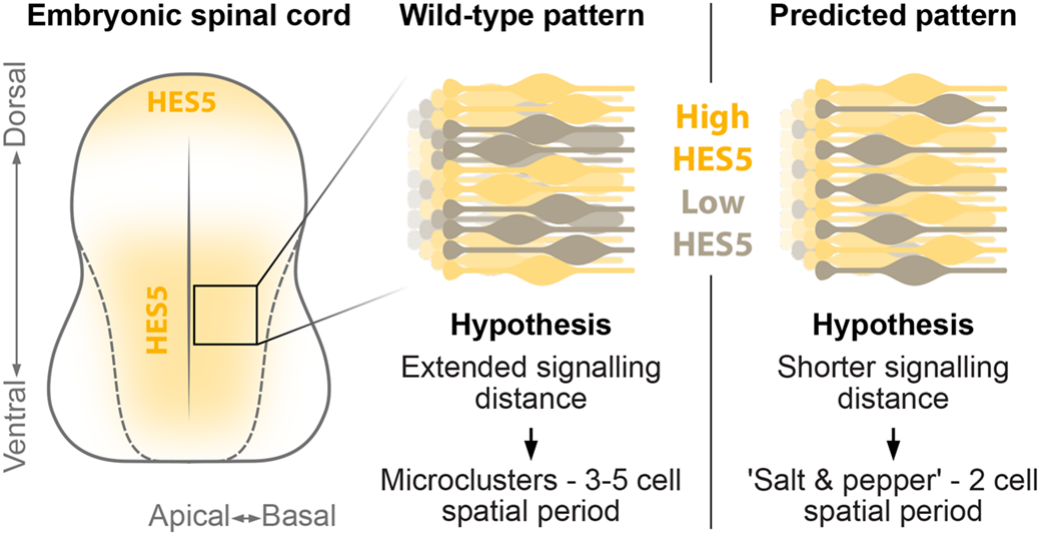
**Overview of HES5 patterning in the embryonic mouse spinal cord.** Left shows the expression regions of HES5 in a cross-sectional view of the spinal cord. Middle shows a magnified illustration of the wild-type HES5 expression pattern in the neuroepithelia that makes up the developing spinal cord. Yellow cells represent higher expressing cells, and grey corresponds to lower expression. In the wild-type neuroepithelia, the expression of HES5 is clustered and has a spatial period of 3-5 cells in the dorsoventral direction. This paper hypothesises that this clustered expression arises from extended Notch signalling distance between cells. Right shows the predicted pattern change to a ‘salt and pepper’ pattern with a shorter spatial period of 2 cells.

However, while Notch signalling is classically thought of as occurring between neighbouring cells, our modelling suggested that longer-range Notch signalling, extending further than immediate neighbouring cells, is necessary for the repetition of HES5 microclusters along the dorsoventral axis with a spatial periodicity of 3-5 cells, as summarised in Fig. 1 (Biga et al., 2021; Hawley et al., 2022). Mathematically, without this long-range signalling, i.e. Notch signals between immediate/adjacent neighbours, a lateral inhibition or ‘salt and pepper’ pattern forms with alternating high-low-high HES5 in adjacent cells (Fig. 1). If signalling distance is extended beyond immediate neighbours this gives rise to the possibility of longer spatial periods between microclusters of cells with correlated HES5 levels and dynamics to form (Hawley et al., 2022).

Indeed, recent findings have shown that long-range signalling events can occur via specialised cellular protrusions, such as cytonemes, that make direct contact with and signal to cells at a distance. Cytonemes can exchange or present signalling molecules between cells and there is increasing evidence for cytoneme regulation of spatial patterning during embryonic development, particularly for the Wnt, Shh and Notch pathways (Bischoff et al., 2013; Cohen et al., 2010; De Joussineau et al., 2003; Hadjivasiliou et al., 2019; Hall et al., 2024; Nelson et al., 2013; Rojas-Ríos et al., 2012; Sagar et al., 2015; Stanganello et al., 2015).

In the Notch pathway, several examples of cytonemes containing Notch/Delta signalling molecules have been reported. In the *Drosophila* notum the regular spacing of sensory organ precursors, which give rise to mechanosensory bristles, require the presence of Notch signalling protrusions that extend beyond neighbouring cells to achieve the observed spacing between cells, and the expression of Delta promotes the formation of these protrusions (Cohen et al., 2010; Corson et al., 2017; De Joussineau et al., 2003). In developing neural tissues DLL1 containing protrusions have also been observed. In the zebrafish spinal cord, differentiating neurons transiently extend long basal protrusions in the anterior-posterior direction to deliver long-range lateral inhibition, and control the spacing of subsequent differentiation events (Hadjivasiliou et al., 2019). In mouse cortex neuroepithelia, protrusions containing DLL1 were found in both radial glial cells (RGCs) and intermediate neural progenitors; however, no functional role for these protrusions was tested (Nelson et al., 2013). In chick spinal cord, actin-dependent protrusions were observed at the apical endfeet of RGCs, though their function was unclear (Kasioulis et al., 2022). Protrusions in the mouse spinal cord were recently observed to emanate from floorplate and roofplate cells carrying Shh and Wnt signals and were required for correct dorsal-ventral patterning (Hall et al., 2024).

Although cellular protrusions have been observed in the mouse spinal cord it is unclear whether they are restricted to floorplate and roofplate signalling centres and the apical endfeet of cells. The latter question is pertinent to the organisation of the neuroepithelium, where neural progenitor cells are bi-polar and elongated, extending the full apicobasal dimension of the spinal cord.

Here, we have used a live membrane reporter and sparse labelling to examine the presence of cellular protrusions in the embryonic mouse spinal cord. We report that actin-based cellular protrusions exist throughout the apicobasal dimension of the radially elongated progenitor cells. Both fixed and live spinal cord slices were imaged to assess protrusion length and we observed protrusions capable of extending contact distance beyond their immediate neighbours. We also found that these protrusions are highly dynamic with an average lifetime of 4 min and contain the Notch signalling ligand DLL1. The protrusions are initiated through CDC42-dependent actin polymerisation as CDC42 inhibition by ML141 resulted in a change in protrusion density but not a reduction in protrusion length. Finally, altering the protrusion density resulted in a tendency towards smaller HES5 spatial periods (reduced by 20% as measured by Fourier transform), as predicted by our previous mathematical modelling, although the change was not statistically significant. This suggests that reducing protrusion density is not sufficient to reveal a contribution to patterning.

## RESULTS

### Protrusions are present in neural progenitor cells of the mouse spinal cord

**Fig. 2.**
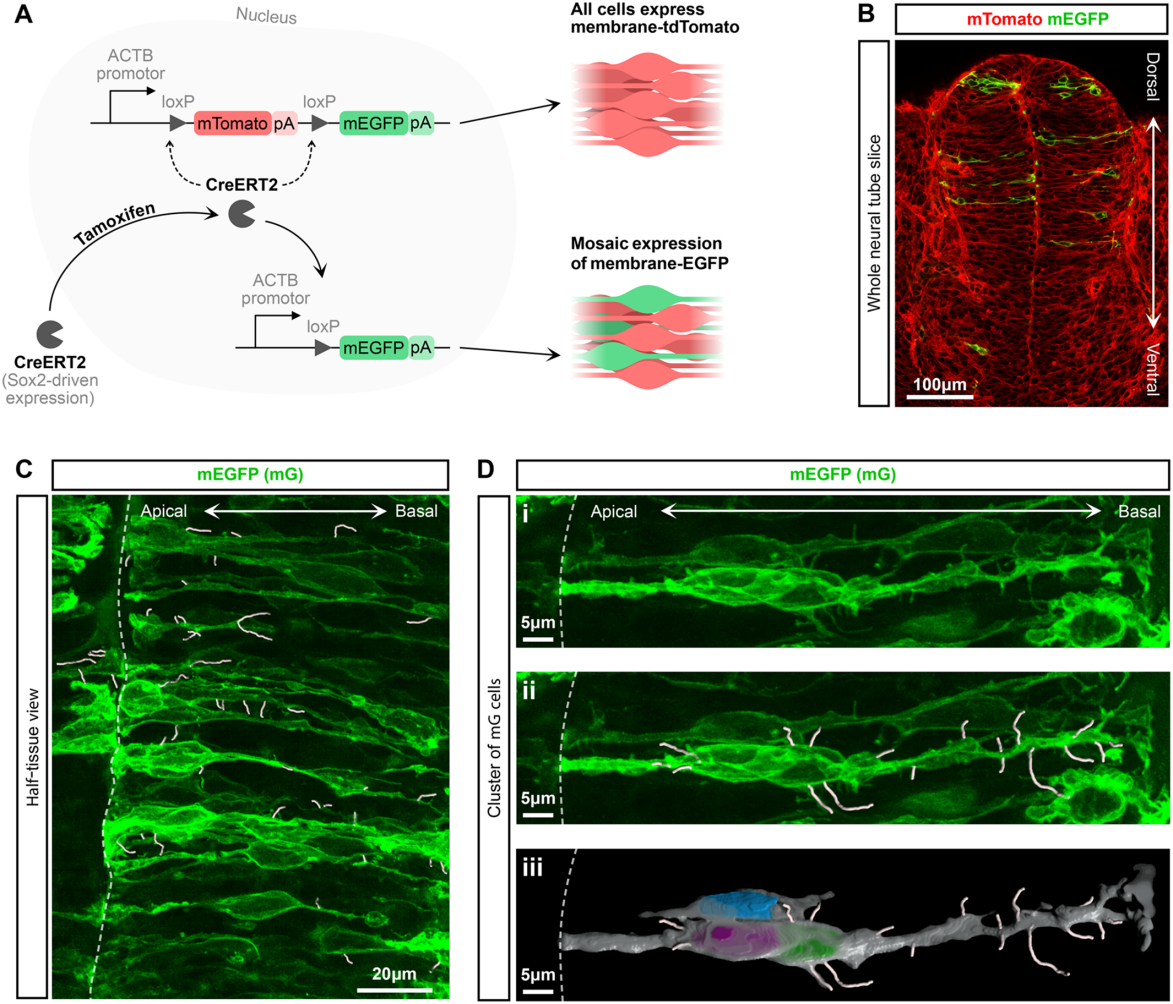
**The Sox2CreERT2 mTmG system is used to mosaically label the membranes of neural progenitor cells in the spinal cord.** (A) Schematic of the Sox2CreERT2 mTmG system: CreERT2 refers to Cre recombinase, which targets and excises genes located between two loxP sites and also has an estrogen ligand-binding domain that requires tamoxifen for it to be localised to the nucleus. The ACTB promotor ubiquitously expresses the downstream genes. pA is the abbreviation for polyA tails. mTomato is membrane-targeted tdTomato fluorophore, and mEGFP is membrane-targeted EGFP. See Materials and Methods 1.1 for further details. (B-D) Confocal images of fixed Sox2CreERT2^+/−^ mTmG^+/−^ E10.5 mouse embryos (Materials and Methods 1.1 and 1.2). (B) Single z-slice image of a whole spinal cord slice showing membrane-tdTomato (mT) in red and tamoxifen-induced membrane-EGFP (mG) in green (see also Movie 1). (C) A z-projection of one side of a spinal cord slice with protrusions traced in white (Materials and Methods 1.5). D shows a magnified view of a group of RGCs (see also Movie 2). Di shows the raw data, Dii shows the data with traced protrusions in white. Diii shows a 3D fill of the membrane (grey) and nuclei of the cells (blue, magenta, green). Separate embryos were used to generate each panel image.

To examine the existence of protrusions in mouse spinal cord RGCs, we used Sox2-tamoxifen-dependent Cre-mediated (Sox2CreERT2) recombination of the membrane-EGFP membrane-tdTomato (mTmG) reporter (Fig. 2A) (Snyder et al., 2013). We used a low dose of tamoxifen to achieve mosaic membrane-EGFP labelling of SOX2 positive cells, allowing individual cell membranes to be distinguished in the densely packed neuroepithelium (Fig. 2B; Movie 1). The bipolar morphology of neural progenitor cells is clearly seen with cellular processes contacting both apical and basal surfaces (Fig. 2B). The higher magnification images in Fig. 2C,D and Movie 2 reveal the presence of thin filopodia-like protrusions on most of these elongated cells, emanating from anywhere along the apicobasal length of the cell. Protrusions were traced and are highlighted with white lines in most panels in Fig. 2C,D. Therefore, the mTmG Sox2CreERT2 system proved sufficient to proceed with both fixed and live imaging to measure and characterise protrusions in mouse neuroepithelia.

### Protrusion lengths and angles in fixed tissue

To understand how far protrusions extend the contact distance in RGCs, both fixed and live spinal cord slices were used to characterise protrusion lengths, angles, and temporal dynamics (see Materials and Methods 1.5).

**Fig. 3.**
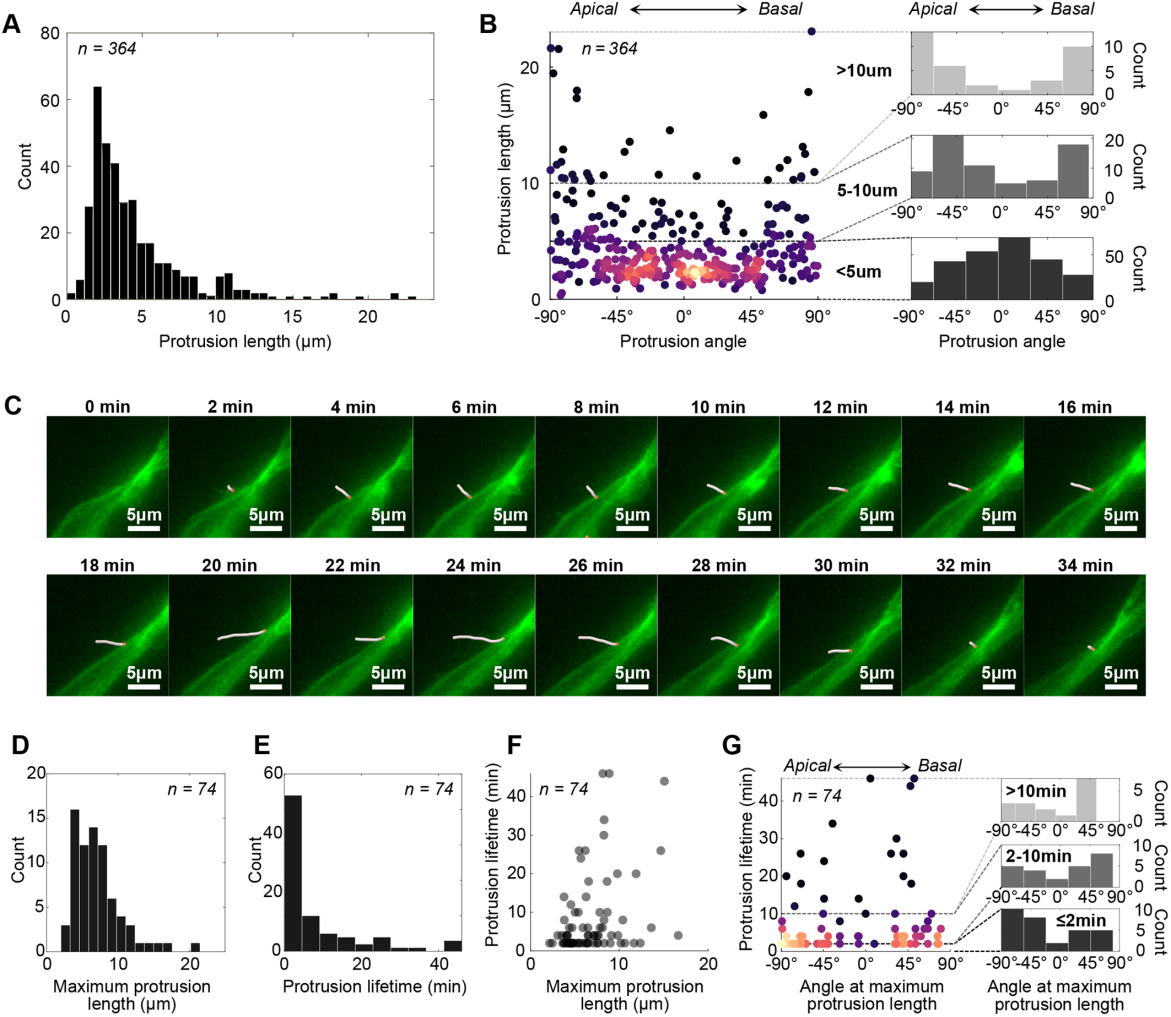
**Quantitative characterisation of protrusion length, angle, and dynamics in both fixed and live tissue.** (A) Histogram of protrusion lengths measured in 12 fixed slices from five different E10.5 Sox2CreERT2^+/−^ mTmG^+/−^ embryos (*n*=364 protrusions). (B) Angle of the protrusions relative to the apicobasal axis (using the same data set as in A). The colouring of the points indicates plot density with lighter colours being higher density. 0° indicates a protrusion oriented perpendicular to the apicobasal axis, −90° is a protrusion oriented towards the apical surface and 90° is a basally directed protrusion. The smaller histogram plots to the right show how the distribution varies with protrusion length. (C) Frames from live imaging (image captured every 2 min) showing the extension and retraction of a protrusion traced in white (the red dot indicates where the protrusion joins the cell body). Movie 3 shows a timelapse with tracked protrusions. (D-F) Characterisation of live-imaged protrusions (*n*=74 protrusions). A protrusion with an outlier lifetime of 150 min was excluded from the data shown. (D) Distribution of maximum protrusion lengths measured for each protrusion tracked in time-lapse imaging. Average maximum length was 6.5±3.6 µm (median±i.q.r.). (E) Distribution of protrusion lifetimes (excluding one outlier at 150 min lifetime). Average lifetime was 4±7 min (median±i.q.r.). (F) Maximum protrusion length plotted against protrusion lifetime. (G) Angle of protrusions at their maximum length/extension versus protrusion lifetime. Histograms on the right show the distribution of protrusion angles at binned lifetimes, indicated in bold. The colouring of the points indicates plot density with lighter colours being higher density.

In fixed tissue, a range of protrusion lengths was observed (Fig. 3A) with the average protrusion length being 3.4±3.3 µm (median±i.q.r.). Fig. 3A shows a skewed length distribution with some protrusions exceeding 20 µm.

Next, protrusion angles were measured relative to the apicobasal axis where −90° is defined as pointing apically, +90° is pointing basally, and 0° pointing perpendicular from the apicobasal axis (along the dorsoventral axis). Shorter-length protrusions were more likely to be perpendicular to the apicobasal axis, which can be seen in Fig. 3B as a higher density of angles around 0° for protrusions less than 5 µm. In the 5-10 µm and >10 µm range, the distribution becomes spread out, becoming more aligned with the apicobasal axis with increasing length.

The persistence length of filopodia, which is the length at which a polymer such as actin will start to bend, is on the order of 10 µm (Mogilner and Rubinstein, 2005; Wisanpitayakorn et al., 2022). This persistence length, combined with the fact the neuroepithelia is a densely packed tissue, suggests that filopodia may be mostly forming perpendicular to the apicobasal axis, and then bending as they lengthen and are obstructed by neighbouring cells. Indeed, many curved and kinked longer protrusions can be seen in Fig. 2C,D.

### Protrusion dynamics in live tissue

To characterise the temporal dynamics of protrusions we used live imaging of E10.5 *ex vivo* mouse spinal cord slices embedded in collagen (see Materials and Methods 1.4). We traced individual protrusions at every time step (every 2 min), as shown in Fig. 3C and Movie 3. Movie 4 shows a cropped and slowed down version of Movie 3 without filament traces to more clearly visualise the protrusions. From this, we obtained the maximum extension length and average lifetime (Fig. 3D,E). The average maximum extension length was found to be 6.5±3.6 µm, and the average lifetime was 4±7 min (median±i.q.r.). Compared to measurements in fixed tissue, the maximum protrusion length distribution is shifted towards longer lengths, and there is a tendency for longer protrusions to have a longer lifetime (Fig. 3F). The relationship between the angle of protrusions when they reach maximum extension versus lifetime was also investigated (Fig. 3G). Across all protrusion lifetimes, there was a tendency for the angle at maximum extension to be more apically or basally directed. However, there was no significant change in the distribution of angles at different protrusion lifetimes, as shown in the histograms in Fig. 3G. Overall, live imaging allows for a more accurate estimate of how far protrusions can reach and reveals that these protrusions are highly dynamic in their extension and retraction.

### Protrusions can extend contact distance beyond immediate neighbours

**Fig. 4.**
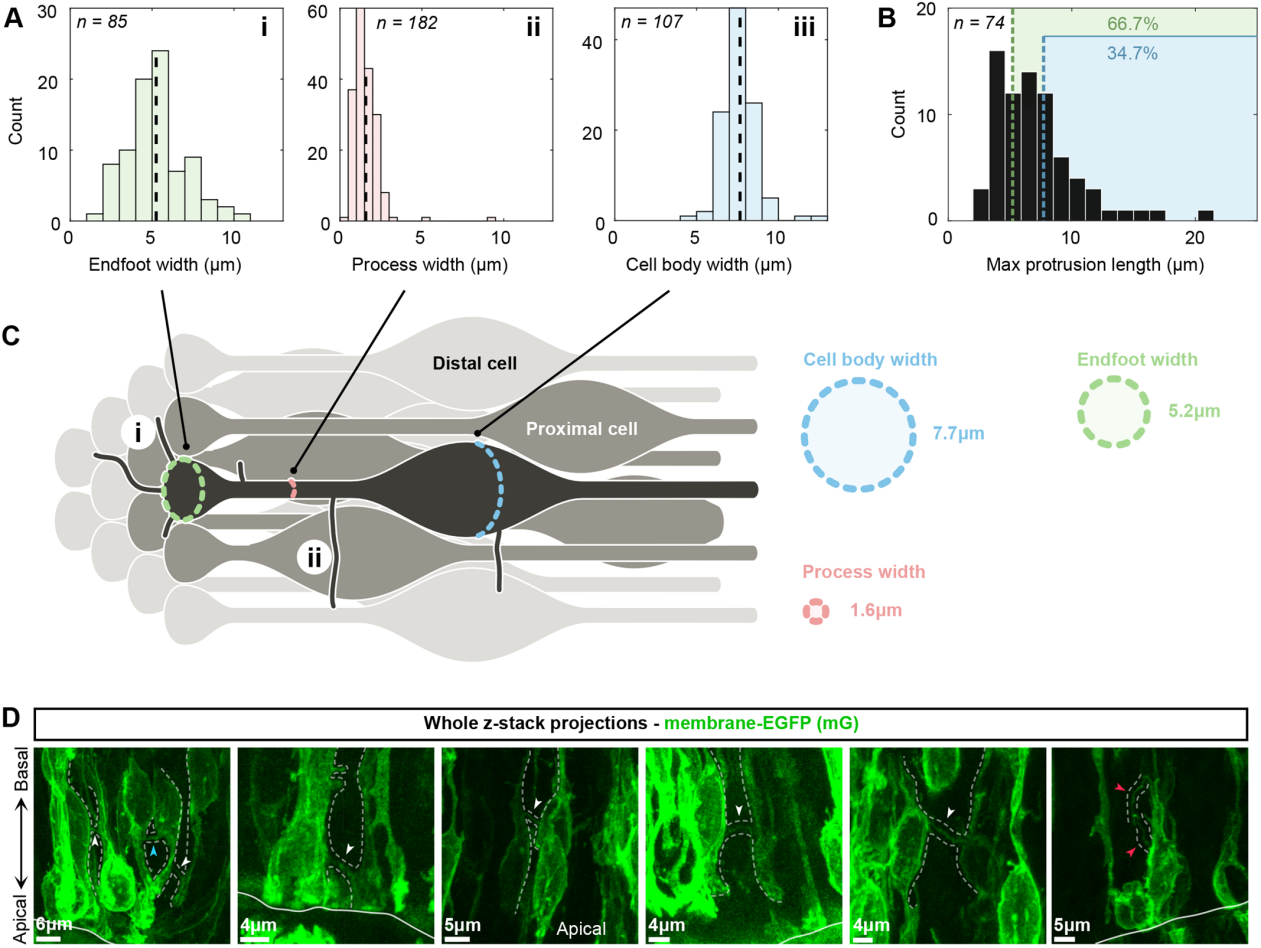
**An exploration of how likely protrusions are to contact non-neighbouring cells.** (A) Width measurements of different parts of RGCs. (Ai) Histogram of apical endfoot widths [*n*=85, average width 5.2±1.8 µm (mean±s.d.)]. (Aii) Histogram of RG process widths [*n*=182, average width 1.6±0.9 µm (mean±s.d.)]. (Aiii) Histogram of cell body widths [*n*=107, average width 7.7±1.1 µm (mean±s.d.)]. See Materials and Methods 1.6 for how protrusions and cell widths were measured. (B) The maximum protrusion length histogram from Fig. 3D (*n*=74), but with the mean measured RGC widths highlighted in different colours. Green is the mean endfoot width (66.7% of protrusions are longer than this), and blue is the mean cell body width (34.7% of protrusions are longer than this). (C) Idealised arrangement of RGCs scaled by the measured cell widths. The cell of interest is labelled in dark grey, proximal cells in mid-grey, and distal cells in light grey. (Ci) A protrusion with a length equal to the mean apical endfoot width, emanating from an apical endfoot. (Cii) Highlights a protrusion with a length equal to the mean RGC cell body width, emanating from a process. (D) Examples of protrusions enabling contacts between cells marked with membrane-EGFP. White arrows highlight protrusions that connect two non-neighbouring/distal cells. Dashed lines indicate the surface of two cells being connected via a protrusion. The blue arrowhead highlights proximal cells making contact via a protrusion. Red arrowheads show an example of two protrusions originating from the same cell contacting each other. Solid white lines highlight the apical surface.

We next wanted to put the measured length distributions into the context of neighbouring and non-neighbouring cells. Therefore, to understand what sort of distance a protrusion would need to traverse to reach a non-neighbouring cell, we constructed a to-scale ‘idealised’ schematic of how RGCs are packed from measurements of three distinct parts of the cell: the apical endfoot width (5.2±1.8 µm, mean±s.d.), the process width (1.6±0.9 µm), and the cell body width (7.7±1.1 µm) (Fig. 4A-C).

A main assumption in the schematic is that the packing of the apical endfeet determines how close together the cells are. Additionally, it assumes that RGCs are straight in the tissue; although our imaging shows they can curve and potentially contact new neighbours along their apicobasal length. This likely results in different cell neighbours along their apicobasal axis, as shown in mouse lung epithelia (Gomez et al., 2021). However, to simplify the interpretation of the protrusion lengths and what is defined as a neighbouring/non-neighbouring cell, we assume that RGCs are straight and not curved or intercalated. In Fig. 4C, we refer to neighbouring cells as ‘proximal’ cells and they are defined as cells whose apical endfeet directly contact the cell of interest (dark grey RGC). Non-neighbouring cells are referred to as ‘distal’ cells. Therefore, this idealised arrangement suggests an RGC has on average a minimum of six neighbours, which is in agreement with the analysis carried out by Guerrero et al. on apical endfeet neighbours (Guerrero et al., 2019).

The location of protrusions on an RGC determines whether a distal cell will be contacted. For example, in Fig. 4Ci, if a protrusion emanates from the apical endfoot, it would need to be longer than 5.2 µm. In this case, 66.7% of protrusions would be long enough to cover this distance (using the maximum extension lengths shown and highlighted in green in Fig. 4B). Alternatively in Fig. 4Cii, if a protrusion emanates from a radial process and needs to cross the width of a cell body (7.7 µm), then 34.7% of protrusions would contact distal cells.

Therefore, protrusions are expected to be capable of making distal contacts, and these were indeed observed in Sox2CreERT2 mTmG embryos (Fig. 4D). The idealised model highlights how the likelihood of making a distal contact depends on where protrusions originate on an RGC. Additionally, these results imply that the six proximal contacts would be the minimum expected number of neighbours for a given cell if no protrusions are present, though with the complex pseudo-stratified epithelium and current mosaic labelling of cells, it is not possible to directly measure the number of neighbours each RGC has.

### DLL1 is present in neural progenitor protrusions

Next, we carried out immunofluorescent (IF) staining in mTmG Sox2CreERT2 spinal cord slices to see if protrusions contain the Notch ligand DLL1, as this would be required for the distal contacts to mediate Notch signalling.

**Fig. 5.**
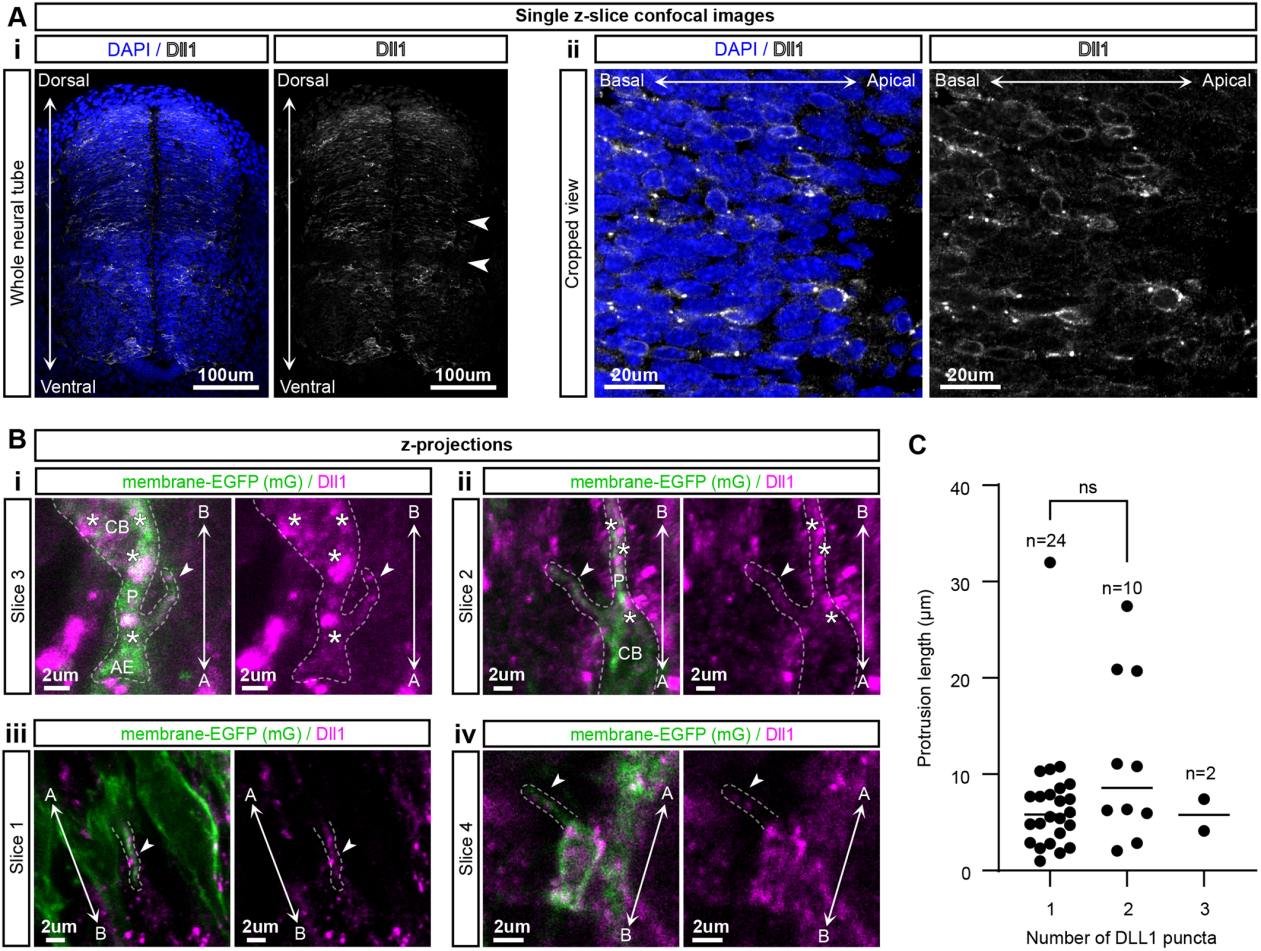
**DLL1 immunofluorescent staining in E10.5 mTmG^+/-^ Sox2CreERT2^+/−^ cryosections (Materials & Methods 1.2).** (Ai) Single z-slice confocal image of a whole spinal cord cryosection showing DAPI and DLL1 immunostaining. The two white arrowheads highlight low expression bands of DLL1 where other ligands are expressed as reported in Marklund et al. (2010). (Aii) Higher magnification images of DAPI and DLL1 from a separate slice to Ai. (Bi-iv) Z-projections of mEGFP and DLL1 where the dashed lines indicate cellular boundaries and white arrowheads highlight protrusions that contain DLL1. Double-headed arrows marked with A and B indicate the apicobasal axis. CB, cell body; P, process; AE, apical endfoot. Asterisks indicate puncta within a cell body or process. (C) Protrusion length for protrusions with 1, 2 or 3 DLL1 puncta that co-localise with the mG membrane fluorescence (Materials and Methods 1.8). A Mann–Whitney test was used to compare the 1 and 2 puncta length distributions, with *P*=0.14. 36 protrusions from 12 slices from four experimental repeats were used.

Fig. 5Ai shows an IF stain for DLL1 (Materials and Methods 1.2). As previously reported, two bands where no DLL1 is expressed, corresponding to the p1 and d6 progenitor domains, were observed (indicated by the white arrowheads) (Marklund et al., 2010). At higher magnification, punctate expression was seen, largely localised to the plasma membrane (Fig. 5Aii). When individual neural progenitor cells were visualised in Sox2CreERT2 mTmG embryos, DLL1 localisation within protrusions was observed (Fig. 5Bi-iv), indicating the possibility that Notch signalling is being transduced via these cellular structures. We also checked if there was a relationship between protrusion length and the number of DLL1 puncta within a given protrusion (Materials and Methods 1.8). We grouped protrusions by the number of puncta present and plotted the distribution of protrusion lengths within each group (Fig. 5C). Most protrusions had just one puncta, and more than two puncta were rare. The length distributions in one and two puncta protrusions showed no significant difference, suggesting longer protrusions do not necessarily contain more puncta. Finally, puncta were not enriched on protrusions; many large puncta are observed in the cell body (asterisks in Fig. 5Bi,ii). This is likely due to the endocytic trafficking that Notch ligands are known to undergo (Nandagopal et al., 2018).

### Protrusions are actin-based and are reduced in density by the CDC42 inhibitor ML141

**Fig. 6.**
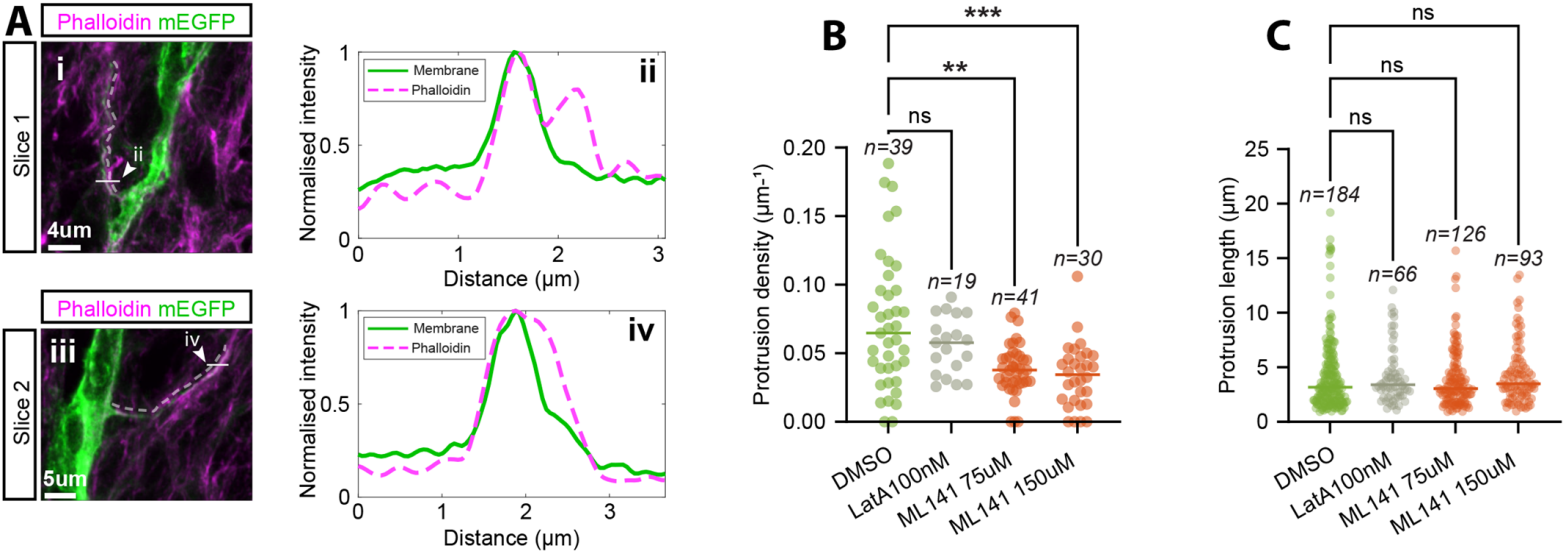
**Evidence that protrusions are actin-based and the effect of actin/filopodia inhibition drugs on protrusion length and density.** (A) Co-localisation of phalloidin in mEGFP marked protrusions in z-projections. Dashed lines highlight the protrusions, and the solid white lines with arrows indicate the line used to generate the intensity profiles in Aii, Aiv. (B,C) Embryos were sliced on a vibratome as in Materials and Methods 1.3, and left for 1 h in DMSO, Latruculin A, or ML141, and then fixed in PFA for imaging. (B) Protrusion density measured in fixed tissue after 1 h of treatment in either DMSO (*n*=39), Latrunculin A 100 nM (*n*=19), ML141 75 µM (*n*=41), or ML141 150 µM (*n*=30) (n-number indicates the number of cells density was measured for) (Materials and Methods 1.5). The statistical test used was a one-way ANOVA on ranks (Kruskall-Wallis) followed by Dunn's multiple comparisons test. *P*-values from left to right: >0.99, 0.0019, 0.0003. (C) Protrusion length measured in fixed tissue after 1 h of treatment in either DMSO (*n*=184), Latrunculin A 100 nM (*n*=66), ML141 75 µM (*n*=126), or ML141 150 µM (*n*=93) (n-number indicates the number of protrusions measured) (Materials and Methods 1.5). The statistical test used was a one-way ANOVA on ranks (Kruskall-Wallis) followed by Dunn's multiple comparisons test. *P*-values from left to right: >0.99, >0.99, 0.53.

Signalling cytonemes, a specialised form of filopodia-like protrusions, can be generated through either a cell dislodgement mechanism, where thin tubes form after adjacent cells touch and move apart; or an actin-driven protrusion mechanism. Our live imaging showed active extension and retraction of protrusions indicating an actin-driven protrusion mechanism. To confirm that the protrusions were actin-based we used phalloidin staining. As expected, we observed F-actin enrichment at the apical surface of the mouse spinal cord, but F-actin was also present in protrusions (Fig. 6Ai-iv).

Previous work has shown that actin-based cytonemes are initiated by cytoskeletal regulators such as CDC42 (Watson et al., 2017). Therefore, we tried to alter the length of these protrusions by adding Latrunculin A, to inhibit actin polymerisation and ML141 to inhibit CDC42 in *ex vivo* embryonic spinal cord slices. Low concentrations of Latrunculin A that did not lead to whole tissue disruption had no effect on the protrusion length or protrusion density, as shown in Fig. 6B,C. However, inhibition of CDC42 using ML141 led to a significant reduction in protrusion density (Fig. 6B) as previously reported in other tissues (Fantin et al., 2015; Grönloh et al., 2023), but protrusion length was unchanged (Fig. 6C). 150 µM of ML141 caused no further decrease in protrusion density compared to 75 µM (Fig. 6B), therefore, to minimize off-target effects and toxicity, 75 µM was used for the remaining experiments in the paper. Taken together, this suggests that RhoGTPase CDC42-dependent actin dynamics initiates protrusion formation in mouse spinal cord neural progenitor cells but does not contribute to protrusion extension.

### Reduction in protrusion density does not significantly alter the HES5 spatial period

Our previous modelling suggested that an extended Notch signalling distance, potentially through protrusions, generated spatially periodic HES5 microclusters along the dorsoventral axis (Hawley et al., 2022). The model predicted that reducing the signalling distance would lead to a more lateral inhibition-like high-low-high HES5 pattern in adjacent cells and hence a shorter HES5 spatial period. Therefore, we experimentally tested whether the decrease in protrusion density caused by the addition of the CDC42 inhibitor ML141 would lead to a reduction in the spatial period of HES5 expression in the spinal cord (Fig. 1).

**Fig. 7.**
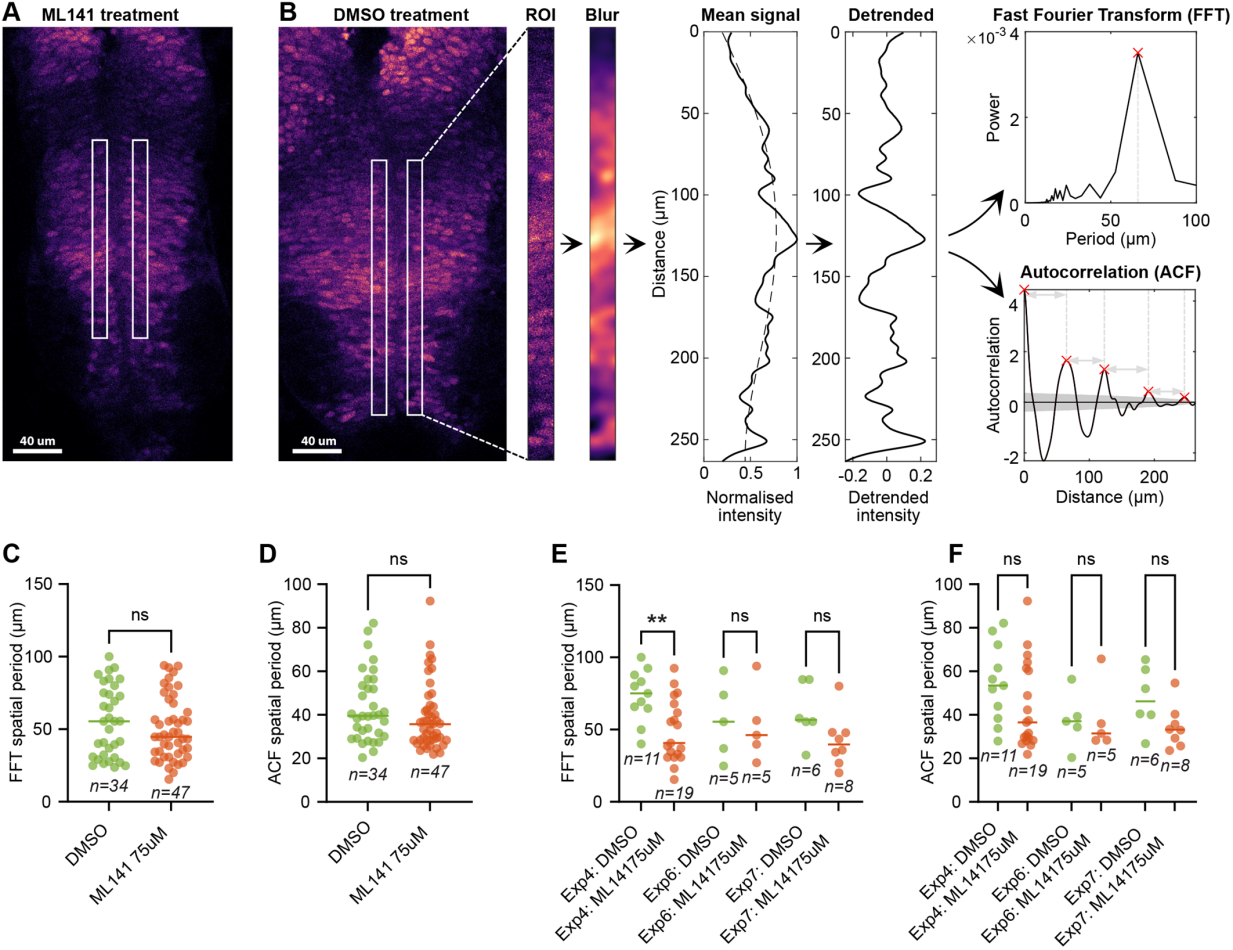
**Spatial periodicity in HES5 expression pattern after DMSO or ML141 treatment.** (A) Venus::HES5 fluorescence in an ML141-treated spinal cord slice (Materials and Methods 1.1). White boxes indicate the regions of interest (ROI) used for spatial period analysis. (B) Analysis pipeline for determining HES5 spatial periodicity along the dorsoventral axis of the spinal cord within the HES5 p0-p2 and pMN domains. The analysis starts with a single z-slice image taken from a confocal z-stack. Next, ROIs are drawn on either side of the ventricle. Then a Gaussian blur with a standard deviation of 3 µm is applied to remove the effects of internuclear periodicity (10 µm). The mean signal is extracted for each row of the ROI, and a polynomial of order 4 (dashed line) is used to detrend. An FFT (Fast Fourier Transform) and ACF (autocorrelation function) method is then used to process the detrended signal. The red cross in the FFT indicates the dominant period and the red crosses in the ACF indicate peaks above the bootstrapping significance threshold visualised as the grey area. Distances between peaks (grey arrows) in ACF are averaged to produce the final spatial period. See Materials and Methods 1.7 for full details of the pipeline. (C-F) Spatial periods of Venus::HES5 fluorescence, where each dot corresponds to an individual ROI from a spinal cord slice. A two-tailed Mann–Whitney *U-*test was used to determine statistical differences in the distributions in all graphs. (C) FFT spatial period of all experimental repeats combined [median values are MedDMSO=55.4 µm (*n*=34), MedML141=4.7 µm (*n*=47), *P*=0.54]. (D) ACF spatial period of combined experimental repeats [median values are MedDMSO=39.6 µm (*n*=34), MedML141=35.7 µm (*n*=47), *P*=0.14]. (E) FFT spatial period of individual experiments that have ≥5 data points – enough for the statistical test (MedExp4:DMSO=75.1 µm (*n*=11), MedExp4:ML141=40.7 µm (*n*=19), *P*=0.0053; MedExp6:DMSO=55.4 µm (*n*=5), MedExp6:ML141=46.2 µm (*n*=5), *P*>0.99; MedExp7:DMSO=56.6 µm (*n*=6), MedExp7:ML141=39.6 µm (*n*=8), *P*=0.0057). (F) ACF spatial period of individual experiments that have ≥5 data points – enough for the statistical test (MedExp4:DMSO=53.4 µm (*n*=11), MedExp4:ML141=36.6 µm (*n*=19), *P*=0.10; MedExp6:DMSO=37.0 µm (*n*=5), MedExp6:ML141=31.5 µm (*n*=5), *P*=0.69; MedExp7:DMSO=46.2 µm (*n*=6), MedExp7:ML141=33.2 µm (*n*=8), *P*=0.081).

Spinal cord slices from embryos with endogenously tagged Venus::HES5 were incubated either in DMSO (control) or 75 µM ML141 for 8 h on an air-media interface and then imaged using a confocal microscope (Materials and Methods 1.3). Slice quality was controlled for, and the left and right sides of the spinal cord were used to extract fluorescence intensity along the dorsoventral axis. This was followed by Gaussian blurring to remove the influence of periodicity from the gaps between nuclei (10 µm periodicity). Detrending was applied to the mean signal to remove the effect of the HES5 domain edges. Finally, the spatial period was estimated either by use of a Fast Fourier Transform (FFT) or autocorrelation function (ACF). An overview of the spatial period analysis pipeline is shown in Fig. 7A,B – full details are given in Materials and Methods 1.7.

Fig. 7C,D show the combined spatial periods from all experimental repeats as calculated by the FFT and ACF methods, respectively. The FFT spatial period in Fig. 7C went from a median of 55.4 µm in DMSO to 44.7 µm in ML141. Likewise, the ACF spatial period in Fig. 7D went from a median of 39.6 µm in DMSO to 35.7 µm in ML141. Neither of these decreases were found to be significantly different. We also examined individual experiment spatial period changes in Fig. 7E,F. This again showed the same tendency for the ML141 condition to have a smaller spatial period compared to DMSO; however, only one experiment showed a significant decrease. In summary, the experimentally measured spatial period of HES5 along the dorsoventral axis had a tendency to decrease when protrusion density was decreased, which agreed with our previous modelling predictions.

## DISCUSSION

Thin cellular extensions are of increasing importance in cell signalling; they have been described to contribute to many of the main developmental signalling pathways such as Notch (Cohen et al., 2010; De Joussineau et al., 2003; Hadjivasiliou et al., 2019; Nelson et al., 2013), Wnt (Hall et al., 2024; Sagar et al., 2015; Stanganello et al., 2015; Zhang et al., 2024), Hedgehog (Bischoff et al., 2013; Hall et al., 2024; Rojas-Ríos et al., 2012), BMP (Hsiung et al., 2005; Inaba et al., 2015; Wilcockson and Ashe, 2019), and FGF (Du et al., 2018).

Here, we have interrogated the presence of cellular protrusions along the apicobasal axis of mouse spinal cord RGCs with a view of testing their potential role in extending Notch signalling distance and driving Notch-dependent gene expression patterning. We have found that such protrusions can reach up to ∼20 µm with a median length of 6.5±3.6 µm (median±i.q.r.). Thus, protrusions can extend contacts beyond neighbouring cells, but the likelihood of this depends upon where protrusions emanate from on a given RGC. Protrusions are also highly dynamic in their extension and retraction with an average lifetime of 4±7 min (median±i.q.r.). While previous findings showed the presence of cellular protrusions in the apical endfeet (Kasioulis et al., 2022) our findings show that they are a lot more extensive as they cover the extent of RGCs along the apicobasal axis.

The protrusions we observed have shared cellular features with cytonemes, a specialised form of filopodia, as they are thin actin-based structures as shown by phalloidin staining and their sensitivity to CDC42 inhibition and contain the signalling ligand DLL1. We describe a CDC42-dependent mechanism for protrusion generation, in contrast to the developing chick limb bud where CDC42 perturbations were not effective in perturbing mesenchymal filopodia formation (Sanders et al., 2013). This indicates that mechanisms driving protrusion and cytoneme formation may be tissue-specific. MyoX is downstream of CDC42 (Bohil et al., 2006) and has recently been shown to regulate initiation of protrusions in mouse spinal cord (Hall et al., 2024). Interestingly MyoX mutants have disrupted Notch signalling as identified by RNAseq (Hall et al., 2024). Further work is required to determine which aspects of the pathway are dysregulated and whether this includes Notch-dependent spatial patterning.

While cytonemes can be involved in local cell-cell signalling, longer-range protrusions which contact non-neighbouring cells can give rise to more complex patterns akin to Turing morphogen reaction-diffusion patterns (Bajpai et al., 2021; Hadjivasiliou et al., 2016; Hawley et al., 2022; Vasilopoulos and Painter, 2016). We hypothesised that protrusions mediate non-neighbouring contacts and we subsequently imaged examples of protrusions extending contact distance beyond immediate neighbours. Further, we found that where protrusions emanate from on RGCs determines how likely non-neighbouring contacts are due to the varying distances they need to traverse.

With few exceptions, the function of protrusions or cytonemes is enigmatic. For example, Kasioulis et al. also inhibited protrusions using truncated WAVE1–eGFP in the chick spinal cord and found that while differentiating neurons reduce their protrusion length it did not affect the number of differentiating cells (Kasioulis et al., 2022). They also found that inhibition of these protrusions did not disrupt neuroepithelium integrity or apical adherens junctions. Our previous mathematical modelling suggested that distant cell contacts mediate the regular spacing of HES5 microclusters with related levels of expression, along the dorsoventral axis of the spinal cord. We furthermore predicted that this spatial HES5 organisation would help even out differentiation events in space by extending Notch/Delta signalling beyond neighbouring cells. Indeed, the presence of DLL1 within the cytonemes reported here suggests that Notch/Delta signalling may occur in these longer-range protrusions. In our mathematical model, the distance between microclusters in the spatially periodic pattern of HES5 is dependent on longer range Notch signalling (Hawley et al., 2022). Without this long range signalling, the spatial HES5 expression would revert to the classical lateral inhibition/‘salt and pepper’ Notch patterning (alternating high and low expressing cells) (Hawley et al., 2022). To probe this prediction and test potential functionality, we perturbed the formation of protrusions and looked at the resulting spatial pattern of HES5.

We initially aimed to alter protrusion length through the actin-disrupting agent Latrunculin A, but this blunt approach led to changes in tissue morphology making it difficult to address spatial patterns of gene expression. At reduced concentrations that maintained tissue architecture, no effect on protrusion length was observed. We do not interpret this to mean that the protrusion length is independent of actin dynamics, rather that the concentrations we used are too low to observe an effect. Therefore, we turned to the CDC42-inhibitor ML141, a more specific inhibitor of actin-based protrusions. This perturbed protrusion density without changing protrusion length, and ultimately we found that lower protrusion density led to a tendency to decrease the spatial period of HES5. However, although the mean spatial period was consistently reduced, the reduction was not statistically significant.

One potential explanation relates to the observation that our experiments ended up altering protrusion density and not protrusion length. It is possible that density changes may not lead to the same changes in spatial patterning as the predicted changes from protrusion length perturbation. Interestingly the Notch-Delta protrusion model constructed by Cohen et al. (2010) found that spatial periodicity of Delta-expressing cells is robust to changes in protrusion signalling likelihood when protrusions are dynamic. If we interpret the signalling likelihood in that model to be analogous to protrusion density, their work may explain why no significant change in the HES5 spatial period was observed when perturbing only protrusion density. More specific perturbations to protrusion length will be required to fully determine if protrusions play a role in the spatial patterning and spatial period of HES5 expression.

An alternative mechanism that could extend cell-cell contact distance is the intercalation of RGCs, which we did not include in our idealised neuroepithelia model. In pseudostratified mouse lung epithelia, Gomez et al. found that cells change neighbours along the apicobasal length due to curvature and changes in cell width, resulting in an increased cell contact distance (Gomez et al., 2021). If it is the case that intercalation extends signalling distance as an alternative mechanism to protrusions, then an intercalated cell would contact different neighbours along its length, resulting in a higher total number of neighbours when compared to a non-intercalated cell. This would imply that the idealised neuroepithelia model would be underestimating how far cells can contact and signal via Notch. Therefore, intercalation plus protrusions would be expected to extend the spatial period of HES5 compared to protrusion alone.

Further work in the spinal cord could define the extent of a cell neighbourhood using a multicolour labelling system such as Brainbow (Dumas et al., 2022; Malaguti et al., 2024) or more agnostic approaches to understand the extent of cell-cell contacts such as the synNotch system (Malaguti et al., 2022; Zhang et al., 2022). Further functional experiments could also be carried out to address how an altered HES5 spatial period affects the patterning of downstream targets. Our previous *ex vivo* experiments and mathematical both predict that altering the spatial patterning would lead to an altered spacing of differentiating cells (Biga et al., 2021; Hawley et al., 2022). This could be tested by observing how the spatial patterning of early differentiation markers (e.g. NGN2, β3-tubulin) change with altered HES5 spatial period.

## MATERIALS AND METHODS

### Mouse lines and tamoxifen administration

Animal experiments were performed within the conditions of the Animal (Scientific Procedures) Act 1986. mTmG (B6.129(Cg)-Gt(ROSA)26Sor^tm4(ACTB-tdTomato,-eGFP)Luo^/J homozygous, strain number 007676) and Sox2CreERT2 (B6;129S-Sox2^tm1(cre/ERT2)Hoch^/J heterozygous strain number 017593) were obtained from The Jackson Laboratory (JAX). Venus::HES5 knock-in mice (ICR.Cg-Hes5<tm1(venus)Imayo>) (Imayoshi et al., 2013) were obtained from Riken Biological Resource Centre, Japan. In these mice the mVenus fluorescent protein is fused to the N-terminus of endogenous HES5. E0.5 was considered as midday on the day a plug was detected. Pregnant mTmG Sox2CreERT2 females were injected with 250 µg or 125 µg (for more sparse cellular labelling) of tamoxifen in 100 µl of corn oil 15-20 h before harvesting the embryos at E10.5. mTmG Sox2CreERT2 embryos of the correct genotype were screened based on the detection of mosaic EGFP expression in the head.

### Immunofluorescence staining

Trunks of E10.5 embryos for cryo-sectioning were fixed in 4% PFA for 1 h at 4°C, followed by three quick washes with 1xPBS and one longer wash for 1 h at 4°C. Embryos were equilibrated overnight in 30% sucrose (MERCK) at 4°C before mounting in Tissue-Tek OCT (Sakura) in cryomoulds and freezing at −80°C. 20-40 µm sections were cut on Leica CM3050S cryostat. Sections were washed twice with PBS. Sections were then blocked in 10% Dako serum-free blocking reagent (Agilent) for 15 min followed by incubation in primary antibody diluted in blocking reagent for 2 h at room temperature or overnight at 4°C. Sections were washed 3×10 min PBS+0.05% Tween-20 (MERCK) washes. Fluorescent Alexafluor-conjugated secondary antibodies were incubated for 1 h at room temperature. Sections were washed 3×10 min PBS+0.05% Tween-20, then incubated with DAPI (ThermoFisher Scientific) and 647-Phalloidin (ThermoFisher Scientific, 1:500) in PBS 5-15 min. Sections were washed twice with PBS and once with H2O before mounting with Moviol 4-88 (MERCK). Primary antibody: sheep anti-DLL1, 1:100 (R&D Systems AF5026). Secondary antibody: Alexafluor 647 anti-sheep, 1:500 (ThermoFisher Scientific). Images were acquired using a Zeiss LSM 880 with a 40×1.30 NA objective.

### Embryo slicing and culture

E10.5 mTmG Sox2CreERT2 embryos were embedded in 4% low-gelling temperature agarose (MERCK) containing 5 mg/ml glucose (MERCK). 200 µm transverse slices of the trunk around the forelimb region were obtained with the Leica VT1000S vibratome and released from the agarose. Embryo and slice manipulation was performed in phenol-red free L-15 media (ThermoFisher Scientific) on ice and the vibratome slicing was performed in chilled 1×PBS (ThermoFisher Scientific). Spinal cord slices were cultured on a 12 mm Millicell cell culture insert (MERCK) with DMEM F-12 media (ThermoFisher Scientific) containing 4.5 mg/ml glucose, 1× MEM non-essential amino acids (ThermoFisher Scientific), 120 µg/ml Bovine Album Fraction V (ThermoFisher Scientific), 55 µM 2-mercaptoethanol, 1× GlutaMAX (ThermoFisher Scientific), 0.5× B27 (ThermoFisher Scientific) and 0.5× N2 (ThermoFisher Scientific) and incubated at 37°C and 5% CO2. Slices were cultured with DMSO or ML141 (MedChemExpress) and fixed in 4% PFA for 1 h. Images were acquired using a Zeiss LSM 880 with a 40×1.30 NA objective.

### Live imaging of spinal cord slices

Spinal cord slices were embedded in type-1 Collagen (Corning) on a glass-bottomed dish (Greiner Bio-one) as described previously (Das et al., 2012). Slices were imaged using a Zeiss Cell Observer Z1 with an environment chamber maintained at 37°C and 5% CO2. Images were acquired with a 40×1.2 NA silicone immersion objective (Carl Zeiss), a Colibri 7 light-emitting diode (LED) light source (Carl Zeiss) and a Flash4 v2 sCMOS camera (Hamamatsu). Image stacks were acquired using minimal exposure times (20 to 50 ms each channel) using intervals of 2 min between exposures.

### Measuring protrusion length, density, and dynamics

The filament tool was utilised in Imaris (Oxford Instruments) in a semi-automatic way whereby automatic filament tracking was skipped and instead, the beginning and end point of the protrusion were defined manually and then the software identified the path of brightest pixels between those two points. This method worked well for sufficiently bright protrusions, and protrusion length was measured as the summed length of each tracked filament. To measure the angle of the protrusions, a reference frame where the y-axis was lined up along the ventricle of the spinal cord, and the x-axis was aligned to the apicobasal direction. From this, the angle from the start and end point was calculated relative to the apicobasal axis and depending on whether it was left or right of the ventricle, angles were corrected so that −90° was towards the ventricle (apical-pointing) and +90° was basal-pointing.

To measure protrusion density, the length of each RGC that was distinguishable from its neighbours was traced, then the number of protrusions associated with that cell was divided by the length of the RGC to get protrusions per µm length of the cell.

### Cell width measurements

The measurement tool in Imaris (Oxford Instruments) was used to determine the width of apical endfeet, processes, and cell bodies. For the apical endfeet, the widest part of the endfoot bulge in the apicobasal direction was measured. The width of processes varies along the apicobasal axis, and so multiple measurements were made at intervals of roughly every 10 µm along the apicobasal axis, meaning each cell contributed multiple process width measurements to the data set. Measurements were only made of processes that were distinguishable from other cells marked with mG. Finally, the cell body width was measured at the widest point perpendicular to the apicobasal axis.

### Spatial period analysis pipeline

Images were processed using Matlab (code and data available https://github.com/Papalopulu-Lab/Hawley2024). Each spinal cord Venus::HES5 image was composed of multiple z-stacks. From each image, an ROI was extracted at every 10 µm apart in the z-depth until the intensity became too low to distinguish Venus fluorescence from background easily. Once these images were loaded into Matlab, boxes 15 µm wide were drawn along the ROI of 15 µm were drawn along the dorsoventral axis as close to the ventricle on each side of the ventricle. The quality of slices was determined by visual inspection of the ROI; sufficiently bright venus signal compared to the background signal and no gaps from the presence of blood vessels or damaged tissue.

Next Gaussian blurring was applied to the ROI signal using a standard deviation of 3 µm. This value was chosen as it was sufficient to remove any spatial periodicity less than 10 µm, which is the distance between neighbouring cells (Biga et al., 2021). The calibration curve for the Gaussian blur standard deviation, along with a comparison of pipeline results with and without blurring is shown in Fig. S1.

The mean signal was then taken per row (in the apicobasal direction) to produce an average dorsoventral signal. This was followed by detrending using polynomial detrending with a polynomial value of 4 (inbuilt polyval and polyfit functions in Matlab).

Finally, the detrended signal was run through either a FFT transform or ACF methods. The FFT used the fft function in Matlab to produce power spectra, the peak period (the frequency with the highest power) was selected as the reported spatial period for that slice. A Fisher g-test was also carried out on each peak period to ensure it was statistically valid (as previously used in Hawley et al., 2022).

For the ACF, the xcorr function in Matlab was used, and then the average distance between peaks of the ACF were used to determine the spatial period. Bootstrapping was applied to determine a statistical cutoff for the peaks, to ensure that they were above noise (previously described in Biga et al., 2021).

### Counting DLL1 puncta

Protrusions that had co-localisation of mG and DLL1 immunofluorescence were identified, and then the number of puncta were counted and assigned to each protrusion. The criteria for a puncta was that it needed to be above background and appear as a well-separated spot, rather than diffuse DLL1 staining.

### Statistical tests

All statistical analysis was carried out using GraphPad Prism. For Fig. 7C-F, a two-tailed Mann–Whitney *U*-test was used. For Fig. 5D,E, a non-parametric one-way ANOVA on ranks (Kruskal-Wallis) followed by Dunn's multiple comparisons test was used. Significance asterisks correspond to: ns, *P*>0.05; ∗, *P*≤0.05; ∗∗, *P*≤0.01; ∗∗, *P*≤0.001.

## Supplementary Material

10.1242/biolopen.061765_sup1 Supplementary information
