# Supplementary material for: Cellular signalling protrusions enable dynamic distant contacts in spinal cord neurogenesis
Source: Biol Open. 2025 Jan 21;14(1):bio061765. doi: 10.1242/bio.061765 (PMC11789279; doi:10.1242/bio.061765)
Supplement: Supplementary information [file biolopen-14-061765-s1.pdf]

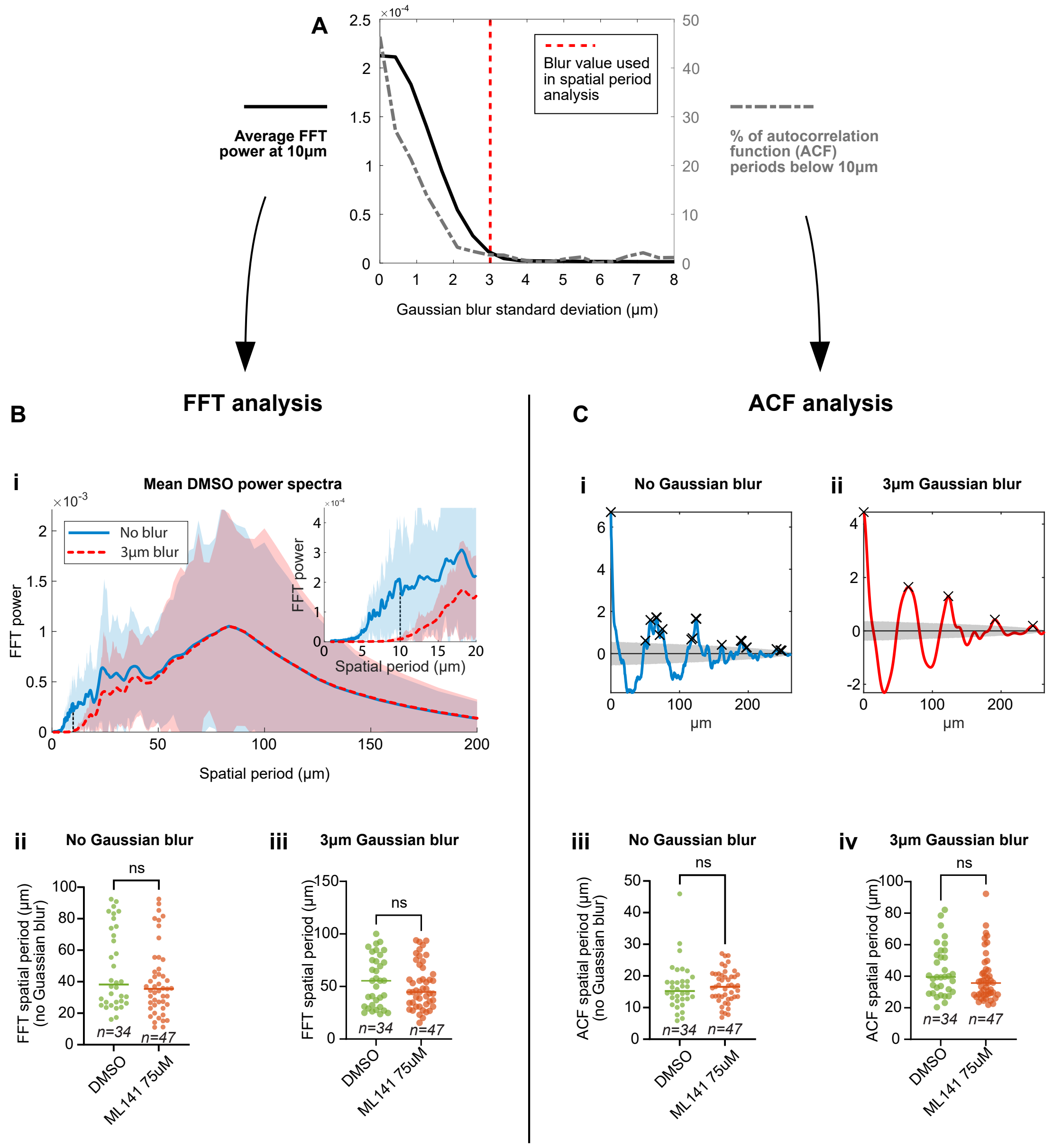

Fig. S1. The effects of using a Gaussian blur step in the spatial period analysis pipeline performed on the Venus::HES5 fluorescence images. A The aim was to remove contributions of 10µm (the internuclear distance as reported in (Biga et al., 2021)) from the spatial periodicity detected by FFT and ACF. The solid black line shows how the average DMSO (all experiments) FFT power at 10µm changes with increasing Gaussian blur standard deviation. Likewise, the dashed grey line shows how the percent of ACF periods below 10um changes with increasing blur standard deviation. The dashed red line at 3µm indicates the blur value used in the paper. **B** Comparison of no Gaussian blur versus using 3µm blur and the effect on the FFT analysis. **B i** The mean DMSO power spectra (all experiments) without blurring applied (solid blue line), and with a blur of 3µm applied (red dashed line). The red and blue error areas are the interquartile range values at each spatial period. The inset graph shows the same graph but zoomed in at the 10µm range. **B ii & iii**, show the FFT spatial periods that are output without and with blurring applied. **C** Comparison of no Gaussian blur versus using 3µm blur and the effect on the ACF analysis. **C i** An example ACF plot without Gaussian blur, where crosses indicate detected peaks above bootstrapping cutoff (grey area). **C ii** The same ACF graph but with 3µm blur applied to the Venus::HES5 fluorescence image. **C iii & iv** show the ACF spatial periods that are output without and with blurring applied.

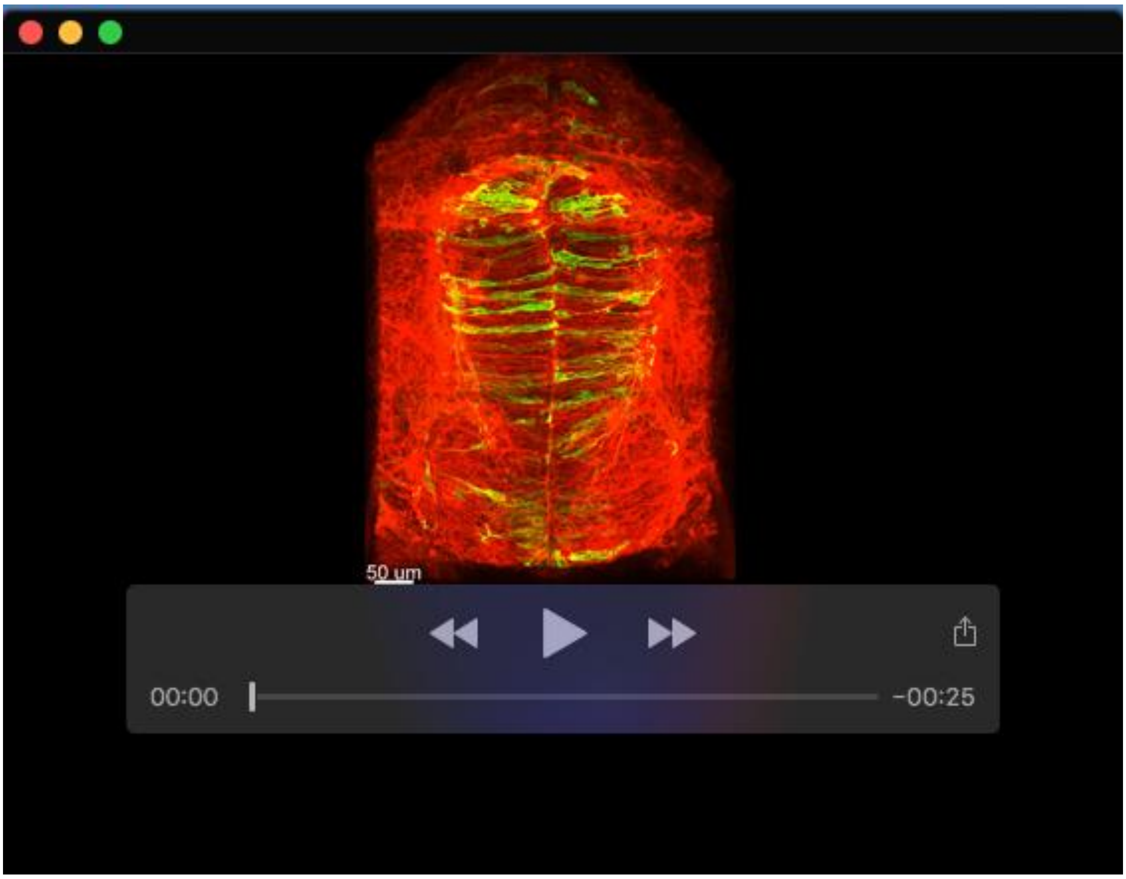

Movie 1. A 360° view of a whole E10.5 mouse embryo spinal cord slice. Confocal z-stack of fixed Sox2CreERT2$^{+/-}$ mTmG$^{+/-}$. Membrane-tdTomato is in red and tamoxifen-induced membrane-EGFP is in green. The slice shown here is the same as in Fig. 2B.

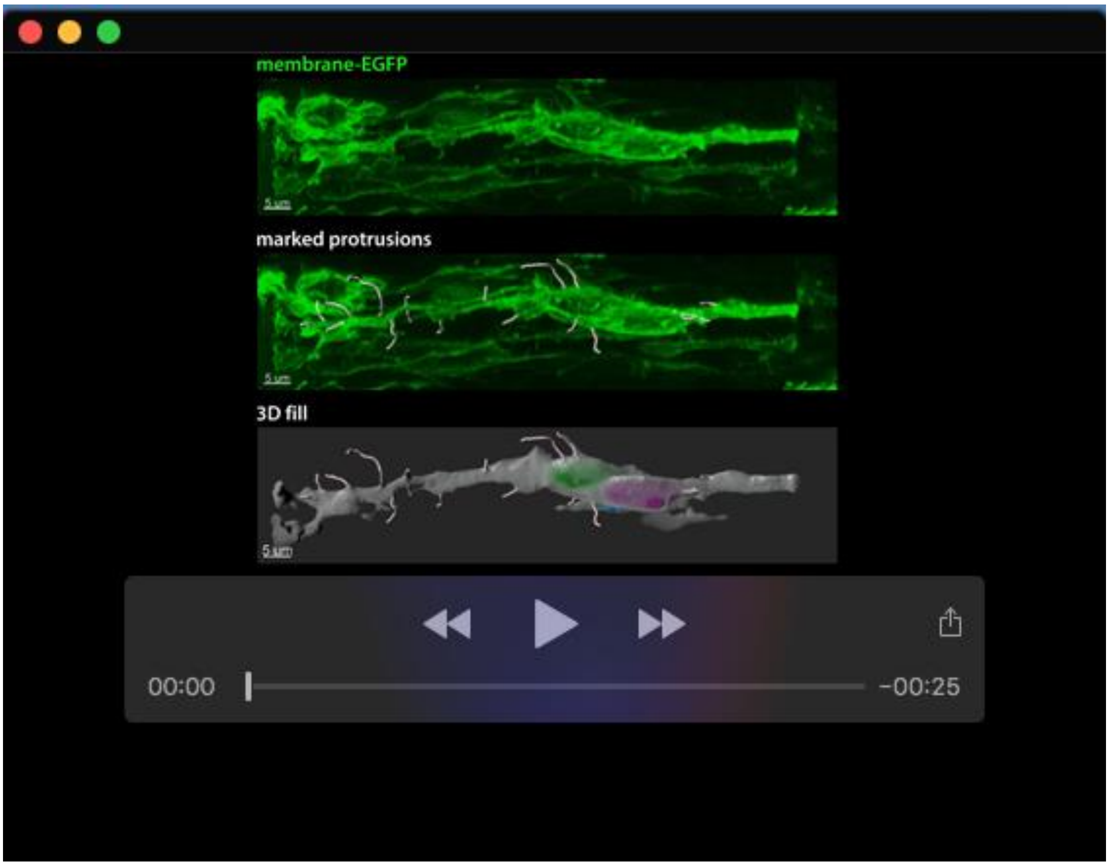

Movie 2. A high magnification 360° view of a confocal z-stack of fixed Sox2CreERT2$^{+/-}$ mTmG$^{+/-}$ E10.5 RGCs. The top panel shows the raw membrane-EGFP fluorescence. The middle panel shows membrane-EGFP with marked protrusions (white) overlayed on the central cluster of 3 cells. The bottom panel shows a 3D surface (made using Imaris) of the three central cells, with the three nuclei highlighted in different colours, and with protrusions marked in white.

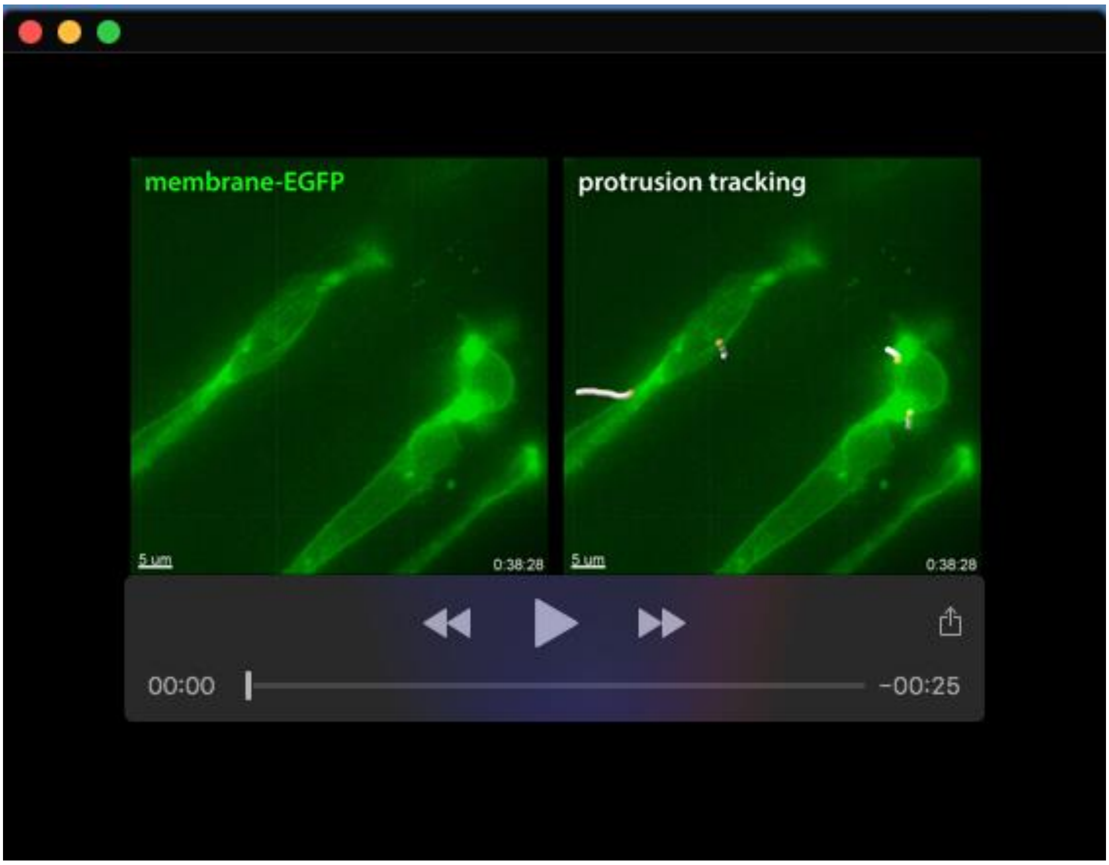

Movie 3. Live imaging of Sox2CreERT2$^{+/-}$ mTmG$^{+/-}$ E10.5 spinal cord slices. The left panel shows membrane-EGFP raw data. The right panel shows the same movie but with tracked protrusions overlayed. Frames were captured every 2 minutes and the time is shown at the bottom right, running to around 4h.

Biology Open • Supplementary information

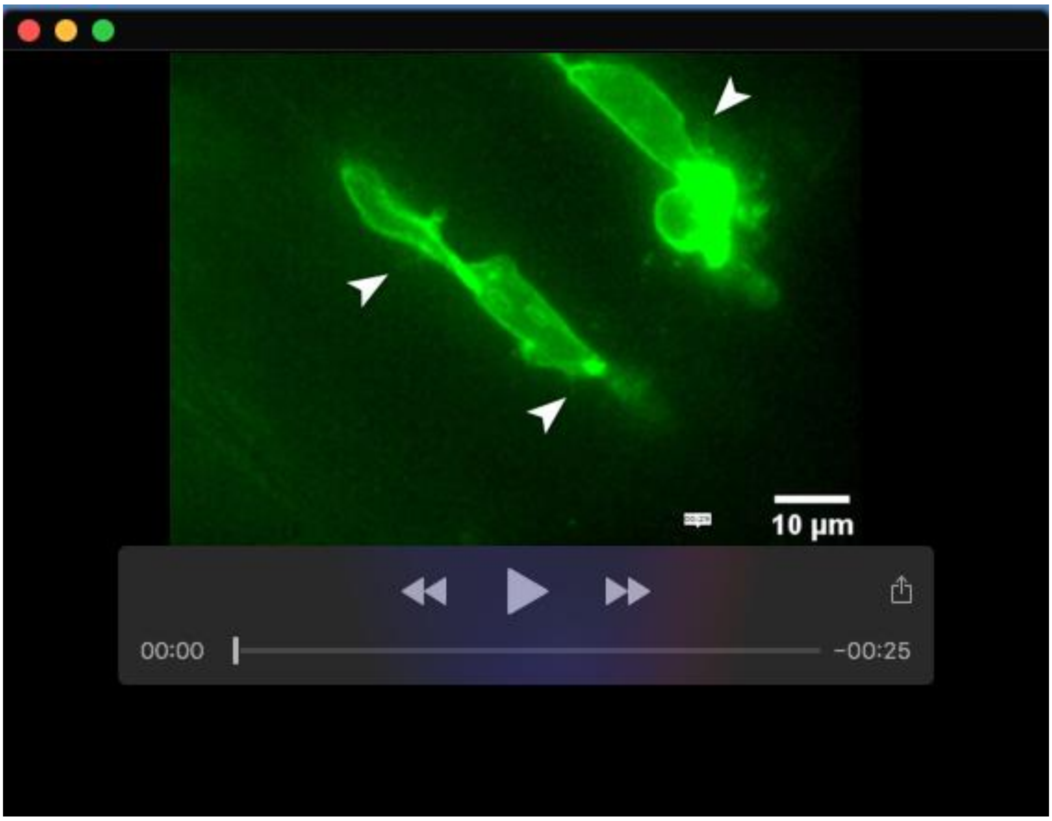

Movie 4. The same data as in Movie 3, but cropped/zoomed in and slowed down to 2 frames per second. The data has been preprocessed with an average 3x3 filter and a sharpening filter of 2x2 to reduce background. Arrowheads are overlaid to highlight protrusions. Frames were captured every 2 minutes.
